# The hymenopteran parasitoid complex (Hymenoptera, Eurytomidae and Pteromalidae) of the weevil beetle *Lixus subtilis* Boheman, 1836 (Coleoptera, Curculionidae) in China

**DOI:** 10.3897/zookeys.1279.188643

**Published:** 2026-05-18

**Authors:** Qin Li, Zhekang Liu, Xiaoyu Li, Huashan Yin, Gerard Delvare, Ekaterina V. Tselikh

**Affiliations:** 1 College of Life Science and Technology, Xinjiang University, 666 Shengli Road, Tianshan District, Urumqi, Xinjiang, 830046, China Université de Montpellier Montpellier France; 2 Xinjiang Key Laboratory of Biological Resources and Genetic Engineering, 666 Shengli Road, Tianshan District, Urumqi, Xinjiang, 830046, China Xinjiang University Urumqi China; 3 CBGP, CIRAD, INRAe, IRD, Montpellier SupAgro, Université de Montpellier, Montpellier, France Zoological Institute, Russian Academy of Sciences St Petersburg Russia; 4 Zoological Institute, Russian Academy of Sciences, St Petersburg 199034, Russia Xinjiang Key Laboratory of Biological Resources and Genetic Engineering Urumqi China

**Keywords:** Description, key, new host, new record, new species, redescription

## Abstract

The hymenopteran parasitoid complex of the families Eurytomidae and Pteromalidae associated with *Lixus subtilis* Boheman, 1836 (Coleoptera, Curculionidae) developing on sugar beet is studied in China (Xinjiang region). *Eurytoma curculionum* Mayr, 1878 (Eurytomidae) was reared from the larvae of *Lixus subtilis* and recorded for Xinjiang, China, for the first time. A new species, *Chlorocytus papahemii***sp. nov**. (Pteromalidae), a hyperparasitoid of *E. curculionum*, is described from China. Illustrations and a detailed morphological description of the new species, as well as a redescription of the types of related *Chlorocytus* species, are provided. An identification key for females of the *Chlorocytus harmolitae* species is given.

## Introduction

The weevil beetle *Lixus subtilis* Boheman, 1836 (Coleoptera, Curculionidae) (Fig. 5) is a harmful pest of agricultural crops, especially sugar beet, in Central and Eastern Europe, Caucasus, Central and Asia Minor, and China. The study of complete parasitic complexes of this weevil beetle, as well as entire food chains including parasitoids and hyperparasitoids, is very limited (Vráblová et al. 2000; Zhang et al. 2017). However, developing biological methods of controlling plant pests should be based on an understanding of real, comprehensive regulatory systems within complete food chains.

**Figures 1–9.**
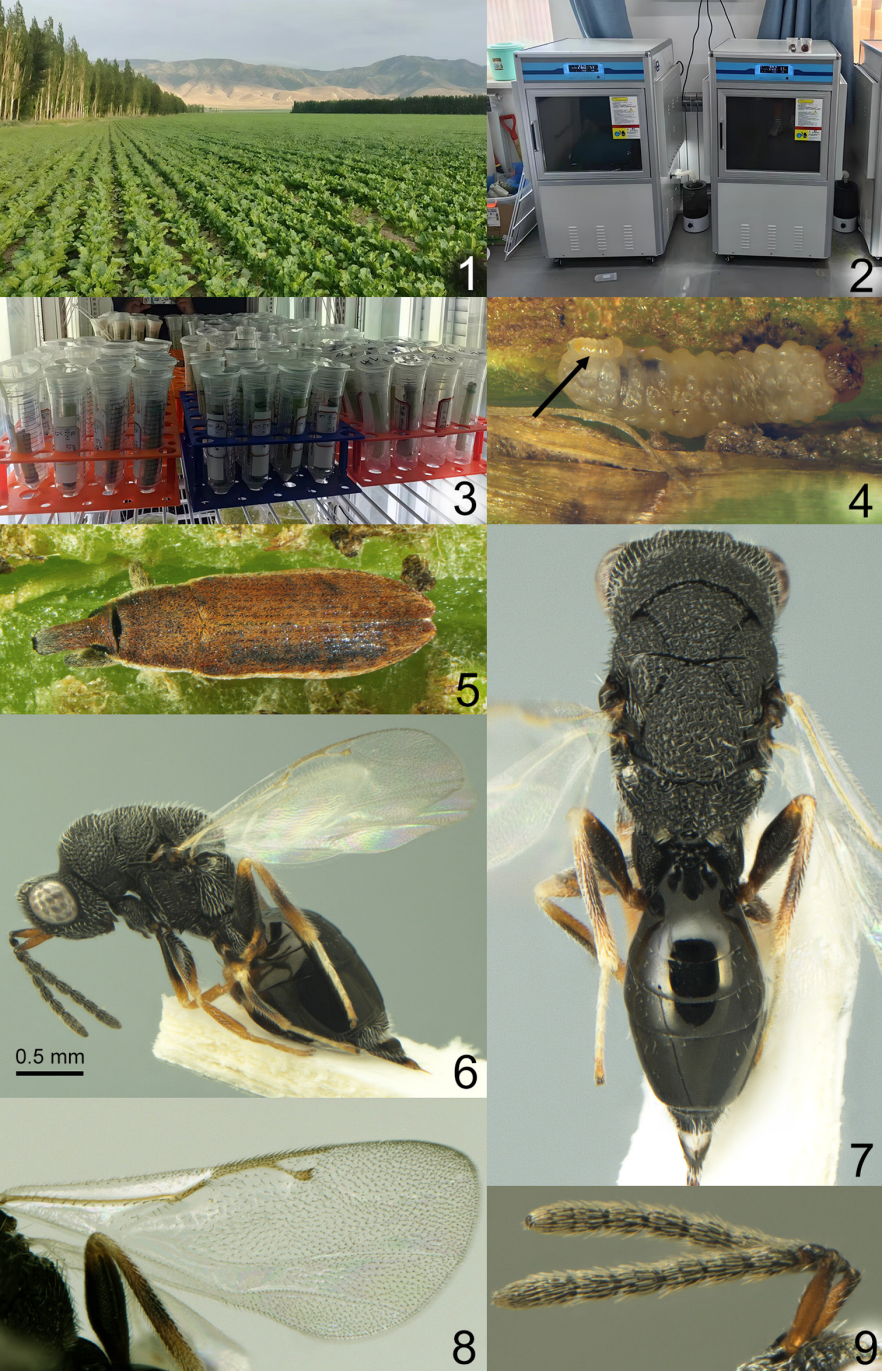
**1**–**4**. Method for rearing parasitoid from infected beetles. **1**. Sugar beet field; **2, 3**. Rearing beetles and parasitoids in a laboratory; **4**. Parasitoid-infected beetle larva (arrow indicates parasitoid larva); **5**. *Lixus subtilis* Boheman, 1836; **6–9**. *Eurytoma curculionum*Mayr 1878 female, not type; **5, 7**. Same, body, dorsal view; **6**. Same, body, lateral view; **8**. Same, fore wing; **9**. Same, antenna.

This paper presents the results of our study of the hymenopteran parasitoid complex from the families Eurytomidae and Pteromalidae of *Lixus subtilis* on sugar beet in China and provides detailed descriptions, illustrations, and an identification key for *Chlorocytus* species.

## Materials and methods

The material (including studied types) used for this study is deposited in the Hymenoptera collections of the Institute of Zoology of the Chinese Academy of Sciences, Beijing, China (**IZAS**), the Zoological Institute of the Russian Academy of Sciences, St Petersburg, Russia (**ZISP**), the Natural History Museum, Vienna, Austria (**NHM**); the College of Life Science and Technology, Xinjiang University, Urumqi, China (**ICXU**), the Science Museum of the Natural Enemies, Geochang, Republic of Korea (**SMNE**), and the Korea National Arboretum, Pocheon, Republic of Korea (**KNA**).

This study was carried out in Xinjiang in China (Yining City and Qapqal Xibe Autonomous County), where *Lixus subtilis* outbreaks have occurred. To obtain the parasitoids associated with the larvae of the pest, sugar beet petioles with traces of *L. subtilis* damage were randomly collected from an agricultural field in this area in August 2024. For this purpose, leaf petioles of sugar beet at least 7–8 cm in length were cut in the field (Fig. 1), transferred to the laboratory, and placed in separate test tubes (Fig. 3). The petioles were dissected to record the number of *L. subtilis* larvae as well as the number of parasitic wasp larvae and pupae (Fig. 4). The beetles and parasitoids were then reared in climate chambers at a temperature of 26 ± 2 °C and 60–70% humidity (Fig. 2).

Morphological terminology, including sculpture and wing venation nomenclature, follows Bouček and Rasplus (1991), Gibson (1997), and Burks et al. (2022). The following abbreviations are used: **POL** – posterior ocellar line, the minimum distance between the posterior ocelli; **OOL** – ocello-ocular line, the minimum distance between a posterior ocellus and compound eye; **F1–F6** – funicular segments; **C1**–**C4** – claval segments; **M** – marginal vein; **S** – stigmal vein; **PM** – postmarginal vein. The scape is measured without the radicle; the pedicel is measured in lateral view. The distance between the clypeal lower margin and the toruli is measured from the lower margins of the toruli. Eye height is measured as its maximum diameter, and eye length as the minimum diameter. The mesosoma and metasoma are measured in lateral view, the latter including the ovipositor sheath.

## Results

New data on biological relationships has allowed us to enhance our knowledge of the biology of parasitoids and study complete parasitic complexes of the *L. subtilis* weevil beetle. *Eurytoma curculionum* (Mayr) was reared from the larvae of *L. subtilis* (Boheman), which feeds on sugar beet, for the first time for China. A new species of pteromalid wasp, *Chlorocytus papahemii* sp. nov., a hyperparasitoid of *E. curculionum*, is described from China. This species belongs to the harmolitae species group within the *Chlorocytus* genus, as detailed in the review below.

### Taxonomy

**Order Hymenoptera Linnaeus, 1758**

**Family Eurytomidae Walker, 1832**

**Subfamily Eurytominae Walker, 1832**

**Genus *Eurytoma* Illiger, 1807**

#### Eurytoma curculionum

Taxon classification

Animalia

HymenopteraEurytomidae

Mayr, 1878

DD383DC8-2D3A-5FD6-8BFF-C5BAE9D2FF03

Figs 6–9

Eurytoma curculionum Mayr, 1878: 301, 306, 314. Lectotype female (NHM, examined).

##### Type material.

***Lectotype*** • female, Austria, “Cleop. camp. Sept. 74”, “*Eur. curculionidarum* [sic] G. Mayr type”, “♀ taken as type Eurytoma curculionum Mayr det. M.F. Claridge .57” (NHM).

##### Additional material examined.

China • 1 female, “Xinjiang, Qapqal Xibe Autonomous County, Aixinshelili Town, Huaxia New Village, sugar beet field, 43°42'43"N, 81°24'48"E, 705 m, 15.VIII.2024, coll. Li Qin research team”, “Host *Lixus subtilis* Boheman, 1836 on sugar beet” (ICXU).

##### Diagnosis.

*Eurytoma curculionum* is very similar to *E. brunniventris* Ratzeburg, 1852 and *E. abrotani* (= *rosae*) (Panzer, 1801) and all these species belong to the abrotani species group due to the following: head with postgenal lamina diverging from the surface of the postgena and with depression step-like margined dorsally; mesocoxa with lamella; propodeum with not impressed median strip. However, *E. curculionum* has a clypeus with the ventral margin hardly or not emarginate and not mesally depressed (vs emarginate and distinctly depressed above emargination), lower part of face rugose-punctate (vs punctate), and anterior outline of mesopleuron ventrally somewhat curved (vs straight).

##### Biology.

*Eurytoma curculionum* is a larval ectoparasitoid of concealed Coleoptera larvae in the families Apionidae, Buprestidae and Curculionidae, as well as of dipterans belonging to the family Cecidomyiidae. It is also a secondary parasitoid of late-instar larvae of *Bracon intercessor* Nees, 1834 (Braconidae) and *Norbanus cerasiops* (Masi, 1922) (Pteromalidae) (UCD Community 2026).

##### Distribution.

Bosnia-Herzegovina, China (new record), Finland, France, Germany, Hungary, India, Iran, Italy, Macedonia, Morocco, Norway, Poland, Sweden, Turkey, United Kingdom (UCD Community 2026).

### Family Pteromalidae Dalman, 1820

**Subfamily Pteromalinae Dalman, 1820**

**Genus *Chlorocytus* Graham, 1956**

#### Chlorocytus papahemii

Taxon classification

Animalia

HymenopteraPteromalidae

Tselikh & Li sp. nov.

FD0CEF7C-B31F-547E-A14C-B86A216DC23B

https://zoobank.org/CBE7B593-53D4-4CB0-8788-40CB21490E21

Figs 24–31

##### Type material.

***Holotype*** • female, China, “Xinjiang, Qapqal Xibe Autonomous County, Aixinshelili Town, Huaxia New Village, sugar beet field, 43°42'43"N, 81°24'48"E, 705 m, 22.VIII.2024, coll. Li Qin research team”, “Host *Lixus subtilis* Boheman, 1836 on sugar beet” (ICXU). ***Paratype*** • female, China, “Xinjiang, Altay Prefecture, Habahe County, 48°3'37"N, 86°23'11"E, 530 m, 14.VII.2020, coll. Li Qin research team” (ICXU).

**Figures 10–16.**
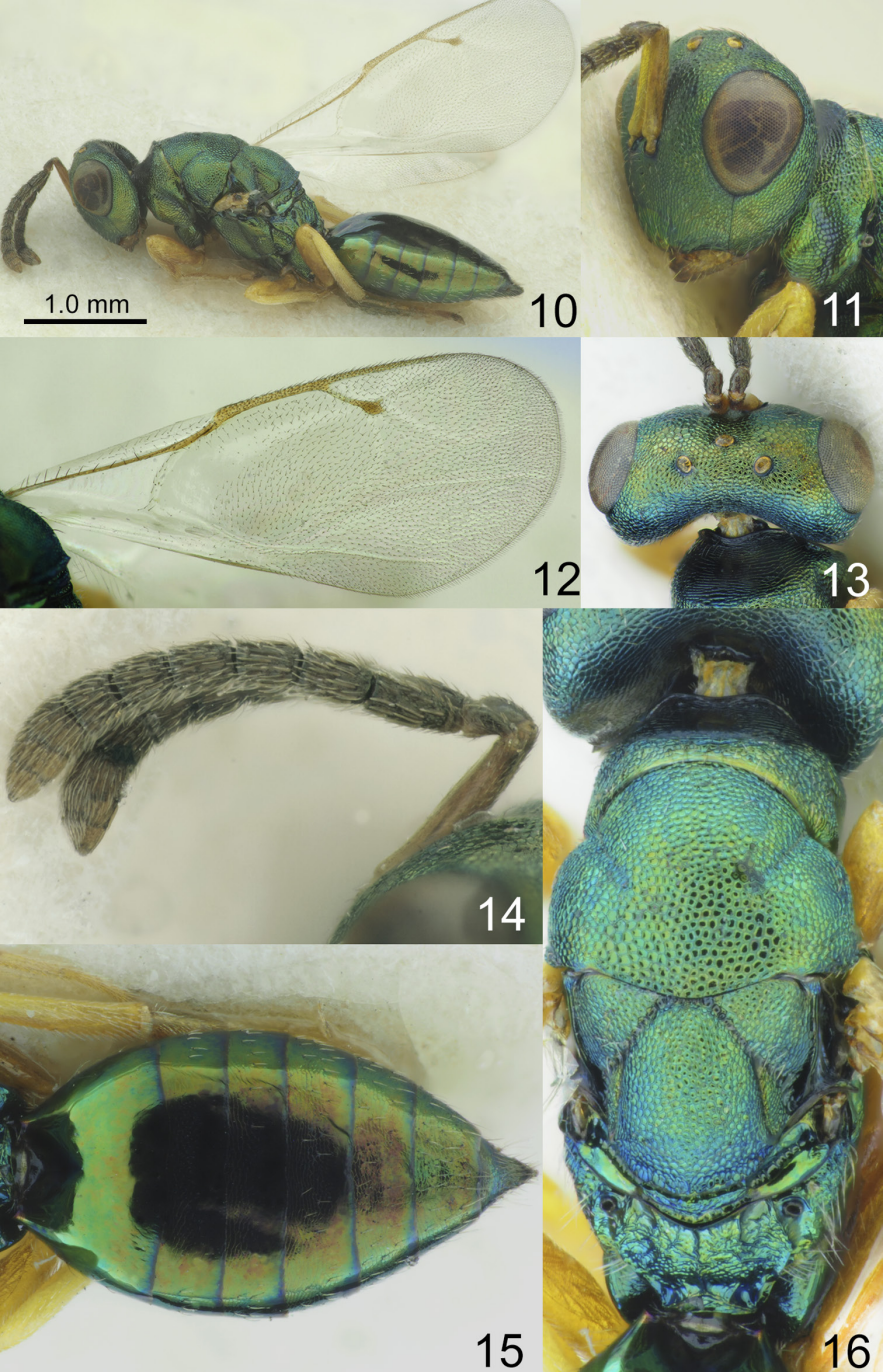
*Chlorocytus harmolitae* Bouček, 1957, female, paratype. **10**. Same, Body, lateral view; **11**. Same, Head, fronto-lateral view; **12**. Same, Fore wing; **13**. Same, Head, dorsal view; **14**. Same, Antenna; **15**. Same, Metasoma, dorsal view; **16**. Mesosoma, Same, dorsal view.

**Figures 17–23.**
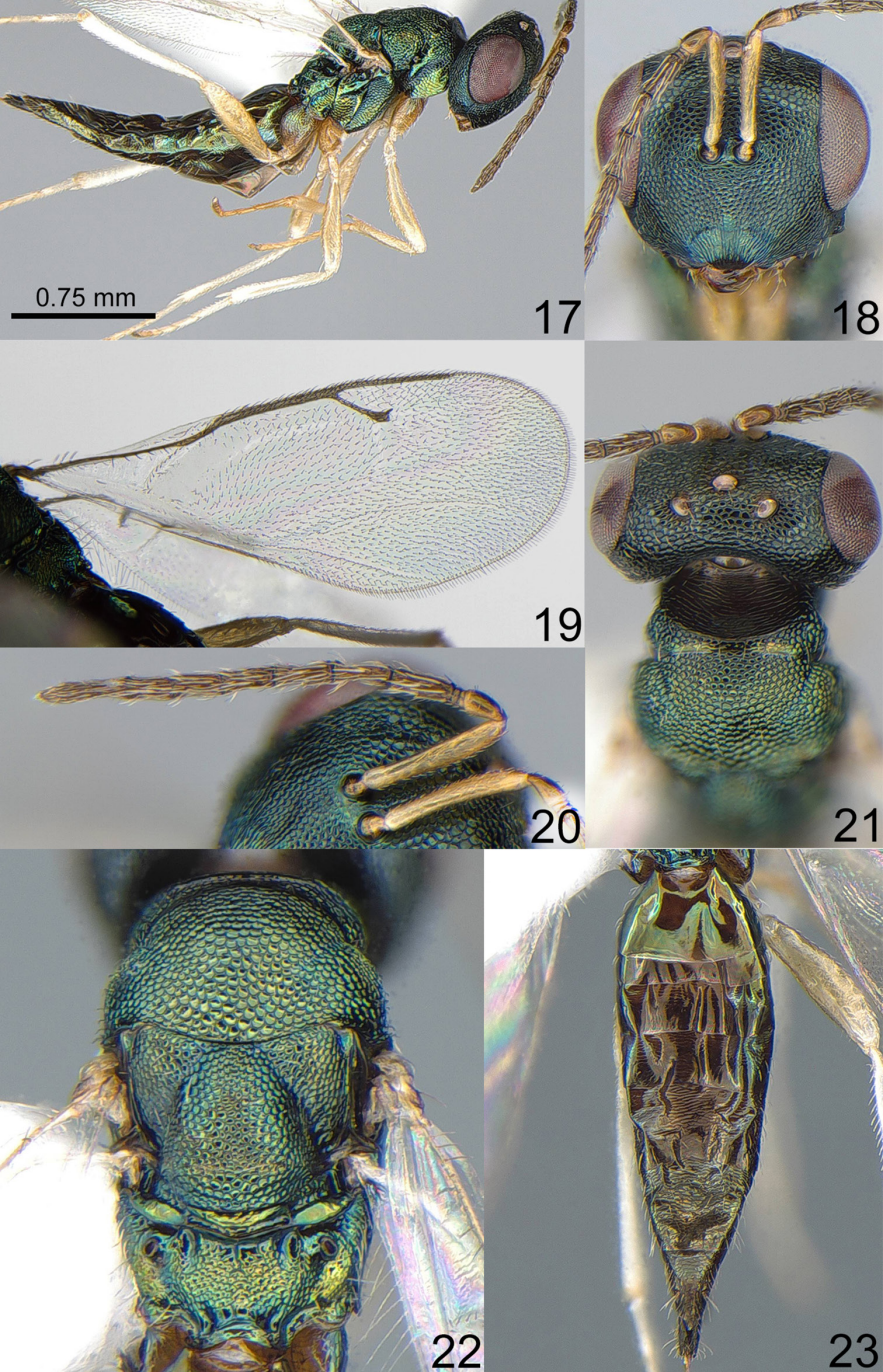
*Chlorocytus jaculatorius* Xiao & Huang, 2000, female, not type. **17**. Same, Body, lateral view; **18**. Same, Head, frontal view; **19**. Same, Fore wing; **20**. Same, Antenna; **21**. Same, Head and pronotum, dorsal view; **22**. Same, Mesosoma, dorsal view; **23**. Same, Metasoma, dorsal view.

**Figures 24–31.**
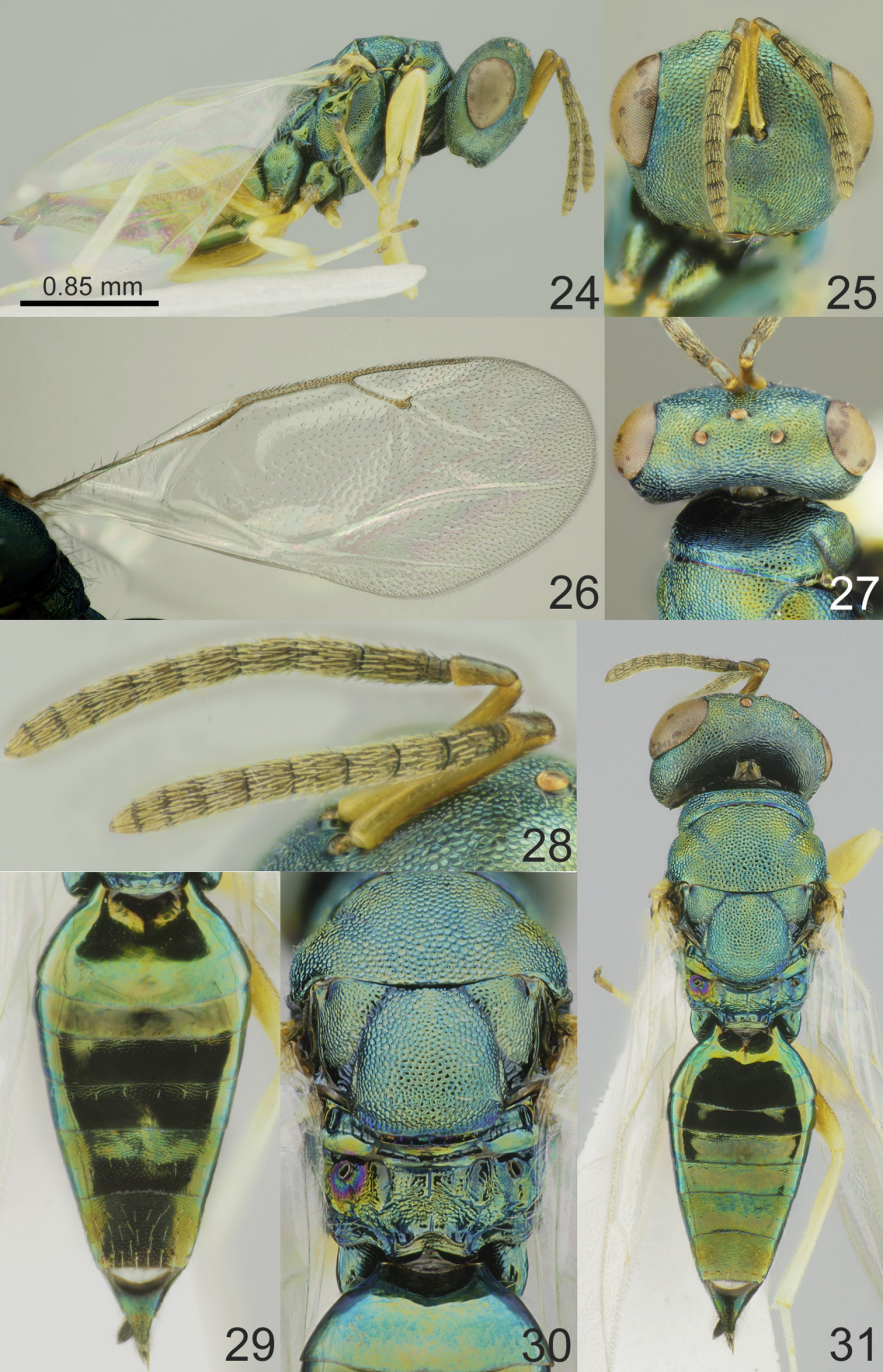
*Chlorocytus papahemii* Tselikh & Li, sp. nov., female, holotype. **24**. Same, Body, lateral view; **25**. Same, Head, frontal view; **26**. Same, Fore wing; **27**. Same, Head and pronotum, dorsal view; **28**. Same, Antenna; **29**. Same, Metasoma, dorsal view; **30**. Same, Mesosoma, dorsal view; **31**. Same, Body, dorsal view.

##### Description.

**Female**. Body length 2.8–3.6 mm; fore wing length 2.24–2.9 mm.

***Colouration***. Head and mesosoma blue-green with metallic, diffuse, coppery lustre; antenna with scape and pedicel yellowish brown; anelli, F1–F6, and clava dark brown. All coxae green with metallic, diffuse, coppery lustre; all femora, tibiae, and tarsi yellow except last segment brown. Fore wing hyaline; venation brown. Metasoma green-blue with metallic, diffuse, coppery lustre; ovipositor sheath brown.

***Sculpture***. Head and mesosoma reticulate; clypeus radially striate; propodeum irregular reticulate; metasoma smooth and shiny.

***Head***. Head in dorsal view 2.39–2.48× as broad as long and 1.23–1.28× as broad as mesoscutum; in frontal view 1.30–1.54× as broad as high. POL 1.42–1.47× as long as OOL. Eye height 1.46–1.50× eye length and 1.85–2.25× as long as malar space. Distance between antennal toruli and lower margin of clypeus near as long as distance between antennal toruli and median ocellus. Antenna with scape 0.95–1.00× as long as eye height and 1.37–1.39× as long as eye length; pedicel 2.00–2.27× as long as broad and 1.05–1.08× as long as F1; combined length of pedicel and flagellum 0.97–1.00× as long as breadth of head; F1 1.87–2.09× as long as broad, with 2 rows of sensilla, F2–F6 longer than broad; clava 2.45–2.50× as long as broad, with small micropilose area on each C3 and C4. Lower margin of clypeus nearly straight.

***Mesosoma***. Mesosoma 1.56–1.60× as long as broad. Pronotal collar with irregular and weak carina. Scutellum arched, 0.94–1.00× as long as broad, frenal area weakly distinct by sculpture. Propodeum 0.56–0.58× as long as scutellum, with costula and irregular median carina. Fore wing 2.24–2.45× as long as its maximum width; basal cell and basal vein bare; speculum open below; M as long as PM and 1.79–1.83× as long as S; stigma small. Mid leg with long spur, more than 0.5× length of first tarsal segment.

***Metasoma***. Metasoma 2.27–2.46× as long as broad, 1.22–1.24× as long as mesosoma, 0.94–1.00× as long as mesosoma and head combined. Petiole transverse. Ovipositor sheath projecting slightly beyond apex of metasoma.

**Male**. Unknown.

##### Etymology.

The species is named in honour of the famous American writer, Ernest Hemingway.

##### Biology.

A hyperparasitoid of *Eurytoma curculionum* (Hymenoptera, Eurytomidae) that develops on the larvae of *Lixus subtilis* (Coleoptera, Curculionidae) in sugar beet leaf petioles, based on the results of observation of larval development.

##### Distribution.

China.

##### Remarks.

This species belongs to the *Chlorocytus harmolitae* species group, which we discuss in detail below.

##### Diagnosis.

Lower margin of clypeus nearly straight (Fig. 25). Antenna with long scape, reaching above median ocellus (Figs 11, 25); F4 longer than broad (Figs 14, 28). POL 1.32–1.54× as long as OOL (Figs 13, 27). Distance between antennal toruli and lower margin of clypeus near as long as distance between antennal toruli and median ocellus. Propodeum 0.42–0.59× as long as scutellum (Figs 16, 30). Fore wing with M 0.96–1.10× as long as PM (Figs 12, 26). All femora, tibiae, and tarsi yellow (Figs 10, 24); mid leg with long spur, more than 0.5× length of first tarsal segment. Metasoma near as long as mesosoma and head (Figs 10, 24).

##### Biology.

Parasitoids of hymenopterans from the family Eurytomidae (Chalcidoidea).

##### Distribution.

Palaearctic region.

### *Chlorocytus harmolitae* species group

**Key to *Chlorocytus harmolitae* species group based on females**

**Table:** 

1	Eye height 2.40–2.55× malar space. Сombined length of pedicel and flagellum 1.33–1.35× breadth of head. Fore and mid coxae yellowish brown (Fig. 17). Propodeum without median carina (Fig. 22). Metasoma 3.05–3.16× as long as broad (Fig. 23) and 1.13–1.15× as long as mesosoma and head (Fig. 17)	***C. jaculatorius* Xiao & Huang, 2000**
–	Eye height 1.85–2.26× malar space. Сombined length of pedicel and flagellum 0.97–1.04× breadth of head. Fore and mid coxae dark green with diffuse, coppery lustre (Figs 10, 24). Propodeum with irregular median carina (Figs 16, 30). Metasoma 1.80–2.46× as long as broad (Figs 15, 29) and 0.77–1.00× as long as mesosoma and head (Figs 10, 24)	**2**
2	Scape 1.11–1.13× as long as eye length; pedicel 1.44–1.6× as long as broad; F4 1.29–1.42× as long as broad; F6 transverse; clava asymmetrical (Fig. 14). Fore wing with basal vein pilose (Fig. 12)	***C. harmolitae* Bouček, 1957**
–	Scape 1.37–1.39× as long as eye length; pedicel 2.0–2.27× as long as broad; F4 1.65–1.90× as long as broad; F6 longer than broad; clava symmetrical (Fig. 28). Fore wing with basal vein bare (Fig. 26)	***C. papahemii* Tselikh & Li, sp. nov**.

#### Chlorocytus harmolitae

Taxon classification

Animalia

HymenopteraPteromalidae

Bouček, 1957

3171263B-FC19-57A6-A71A-4007FB29E0E9

Figs 10–16

Chlorocytus harmolitae Bouček, 1957: 156. Holotype female (NMP, not examined).

##### Type material.

***Paratypes*** • female, Czech Republic, “Bohemia or. Hradec Králové Bouček”, “Piletice 1.VI.52”, “Paratypus ♀ *C. harmolitae* Bčk” (ZISP) • 1 female, Czech Republic, “Moravia centr. VI.48”, “Legit: Prof. Fr. Gregor.”, “*Ch. harmolitae* Bčk. PARATYPUS ♀” (ZISP).

##### Description.

**Female**. Body length 3.65–4.0 mm; fore wing length 2.9–3.2 mm.

***Colouration***. Head and mesosoma dark blue-green with metallic, diffuse, coppery lustre; antenna with scape brown; pedicel, anelli, F1–F6, and clava dark brown. All coxae green with metallic, diffuse, coppery lustre; all femora, tibiae, and tarsi yellow except last segment brown. Fore wing hyaline; venation yellowish brown. Metasoma blue-green with metallic diffuse, coppery lustre; ovipositor sheath brown.

***Sculpture***. Head and mesosoma reticulate; clypeus radially striate; propodeum irregularly reticulate; metasoma smooth and shiny.

***Head***. Head in dorsal view 2.10–2.13× as broad as long and 1.28–1.30× as broad as mesoscutum; in frontal view 1.3–1.36× as broad as high. POL 1.32–1.40× as long as OOL. Eye height 1.24–1.29× eye length and 2.23–2.26× as long as malar space. Distance between antennal toruli and lower margin of clypeus as long as distance between antennal toruli and median ocellus. Antenna with scape 0.85–0.89× as long as eye height and 1.11–1.13× as long as eye length; pedicel 1.44–1.60× as long as broad and 0.69–0.71× as long as F1; combined length of pedicel and flagellum as long as breadth of head; F1 1.70–1.75× as long as broad, with 3 rows of sensilla, F2–F4 longer than broad, F5 subsquare, F6 transverse; clava 1.84–1.90× as long as broad, with small micropilose area on each C3 and C4. Lower margin of clypeus nearly straight.

***Mesosoma***. Mesosoma 1.95–2.00× as long as broad. Pronotal collar with irregular, weak carina. Scutellum weakly arched, 1.17–1.20× as long as broad, frenal area weakly distinct by sculpture. Propodeum 0.45–0.57× as long as scutellum, with costula and irregular median carina. Fore wing 2.36–2.4× as long as its maximum width; basal cell bare; basal vein pilose; speculum open below; M 0.96–1.00× as long as PM and 1.77–1.80× as long as S; stigma small. Mid leg with long spur, more than 0.5× length of first tarsal segment.

***Metasoma***. Metasoma 1.80–1.86× as long as broad, near as long as mesosoma, 0.77–0.80× as long as mesosoma and head combined. Petiole transverse. Ovipositor sheath projecting slightly beyond apex of metasoma.

**Male**. Unknown.

##### Biology.

Primary parasitoid of *Tetramesa eximia* (Giraud, 1863) (Hymenoptera, Eurytomidae) (Bouček 1957).

##### Distribution.

Czech Republic, France, Germany, Netherlands, Romania, Spain, Sweden, United Kingdom (UCD Community 2026).

##### Remarks.

This species is very similar to *C. papahemii* Tselikh & Li, sp. nov.; the differences between these species are given in the key.

#### Chlorocytus jaculatorius

Taxon classification

Animalia

HymenopteraPteromalidae

Xiao & Huang, 2000

EC1177E3-C230-5C6E-A29A-6F07F50DE992

Figs 17–23

Chlorocytus jaculatorius Xiao & Huang, 2000: 311, 315–316. Holotype female (IZAS, examined).

##### Type material.

***Holotype*** • female, China, **“**P. R. CHINA: Ningxia: Jingyuan (35.5°N, 106.3°E), 9.vii.1984, Ding-Xi LIAO”, “HOLOTYPE”, “*Chlorocytus jaculatorius* sp. nov. Xiao & Huang. 2000”, “*Chlorocytus* det. DWH & SLH 1997”, “IOZ(E) 932271”. (IZAS).

##### Additional material examined.

China • 1 female, “Xinjiang, Hami City, Huayuan Township, 42°44'56"N, 93°28'35"E, 664 m., 1.VIII.2012, coll. Hu Hongying” (ICXU). South Korea • 1 female, “Gangwon-do, Mandae-ri, Haean-myeon, Yanggu-Gun, Gangwon-do, 18.VII 2014, coll. H.T. Shin” (KNA) • 1 female, “Gyeonggi-do, Soheul-eup, Pocheon-si, Gyeonggi-do, 37°45'29.2"N, 127°10'0.4"E, 31.VIII–15.IX 2015, coll. Park, Choi, Nam, Shin, Kim” (KNA) • 1 female, “Gyeongsangbuk-do, Daehyeon-ri, Bukhu-myeon, Andong-si, MT, 16.VI–30.VI.2021, coll. Gwon Gimyeon” (SMNE) • 1 female, “Dokdo-ri, Ulleung-eup, Ulleung-gun, swiping, IX.2017, coll. Ku Deokseo, Lee Hyerin” (SMNE) • 1 female, “Hakpo-ri, Seo-myeon, Ulleung-gun, MT, 15.VII–31.VII 2017, coll. Ku Deokseo” (SMNE) • 2 females, “Gyeongsangnam-do, Daedong-ri, Mari-myeon, Geochang-gun, MT, 22.V–15.VI 2020, 15.VI–30.VI 2020, coll. Lee Jaehyeon, Jeong Hyojin” (SMNE).

##### Description.

**Female**. Body length 2.7–3.1 mm; fore wing length 2.1–2.5 mm.

***Colouration***. Head and mesosoma dark blue-green with metallic, diffuse, coppery lustre; antenna with scape and pedicel yellowish brown; anelli, F1–F6, and clava brown. Fore and mid coxae yellowish brown; hind yellowish brown with green metallic lustre; all femora, tibiae, and tarsi yellow. Fore wing hyaline; venation brown. Metasoma brown with metallic diffuse blue, green, and coppery lustre; ovipositor sheath dark brown.

***Sculpture***. Head and mesosoma reticulate; clypeus radially striate; propodeum reticulate; metasoma smooth and shiny.

***Head***. Head in dorsal view 1.95–2.20× as broad as long and 1.32–1.35× as broad as mesoscutum; in frontal view 1.16–1.17× as broad as high. POL 1.47–1.54× as long as OOL. Eye height 1.3–1.35× eye length and 2.40–2.55× as long as malar space. Distance between antennal toruli and lower margin of clypeus as long as 1.00–1.25× distance between antennal toruli and median ocellus. Antenna with scape 0.76–0.78× as long as eye height and as long as eye length; pedicel 1.91–2.00× as long as broad and 0.7–0.74× as long as F1; combined length of pedicel and flagellum as long as 1.33–1.35× breadth of head; F1 2.7–2.73× as long as broad with 2 rows of sensilla, F2–F6 longer than broad; clava 2.56–2.6× as long as broad, with small micropilose area on each C3 and C4. Lower margin of clypeus nearly straight.

***Mesosoma***. Mesosoma 1.86–1.89× as long as broad. Pronotal collar with distinct and strong carina. Scutellum arched, 1.06–1.17× as long as broad, frenal area distinct by sculpture. Propodeum 0.42–0.59× as long as scutellum, with costula and without median carina. Fore wing 2.46–2.65× as long as its maximum width; basal cell bare; basal vein pilose; speculum open below; M 1.00–1.10× as long as PM and 1.81–2.20× as long as S; stigma small. Mid leg with long spur, more than 0.5× length of first tarsal segment.

***Metasoma***. Metasoma 3.05–3.16× as long as broad, 1.36–1.50× as long as mesosoma, 1.13–1.15× as long as mesosoma and head combined. Petiole transverse. Ovipositor sheath projecting slightly beyond apex of metasoma.

**Male**. Unknown.

##### Biology.

Unknown.

##### Distribution.

China, South Korea (Xiao and Huang 2000; Tselikh et al. 2022).

##### Remarks.

This species is very similar to *C. harmolitae* and *C. papahemii* Tselikh & Li, sp. nov.; the differences between these species are given in the key.

## Supplementary Material

XML Treatment for Eurytoma curculionum

XML Treatment for Chlorocytus papahemii

XML Treatment for Chlorocytus harmolitae

XML Treatment for Chlorocytus jaculatorius

## Additional information

### Artificial Intelligence (AI) use

The authors accept full responsibility for the content of the manuscript, including the disclosure of any use of AI.

No AI tools were used in the preparation of this manuscript.

### Author ORCIDs

Q. Li https://orcid.org/0000-0002-1780-7332

X. Li https://orcid.org/0009-0005-3929-9921

H. Yin https://orcid.org/0009-0001-3793-2093

G. Delvare https://orcid.org/0000-0002-6854-2813

E. V. Tselikh https://orcid.org/0000-0002-9184-043X

### Data availability

All of the data that support the findings of this study are available in the main text.
